# The endophytic fungi of *Salvia miltiorrhiza* Bge.f. alba are a potential source of natural antioxidants

**DOI:** 10.1186/s40529-015-0086-6

**Published:** 2015-04-01

**Authors:** Yan-Ling Li, Xiao-Ming Xin, Zheng-Yao Chang, Ren-Jiu Shi, Zeng-Min Miao, Jing Ding, Gang-Ping Hao

**Affiliations:** 1 College of Life Sciences, Taishan Medical University, Tai’an, 271016 China; 2 College of Pharmacy, Taishan Medical University, Tai’an, 271016 China; 3 College of Chemical Engineering, Taishan Medical University, Tai’an, 271016 China

**Keywords:** Salvia miltiorrhiza Bge.f.alba, Endophytic fungi, Identification, Phytochemicals, Antioxidant activity

## Abstract

**Background:**

*Salvia miltiorrhiza* Bge. f. alba is a traditional Chinese herbal drug with special pharmacological effect on thromboangiitis obliterans. However, the nature source of *S.miltiorrhiza* Bge.f.alba is now in short supply because of the over-collection of the wild plant. To better utilize this resource, the diversity and antioxidant activity of endophytic fungi isolated from *S. miltiorrhiza* Bge. f. alba were investigated.

**Results:**

A total of 14 endophytic fungi were isolated from different parts of *S. miltiorrhiza* Bge.f.alba. Based on morphological and molecular identification, the endophytic fungi isolated were classified into four genera (*Alternaria* sp*., Fusarium* sp., *Schizophyllum* sp*.* and *Trametes* sp*.*). These fungal extracts were prepared using ethanol and evaluated for their phytochemical compounds and antioxidant activity. *Alternaria alternata* SaF-2 and *Fusarium proliferatum* SaR-2 are of particular interest because they yielded all of nine phytochemicals including saponins, phenol, flavonoids, cardiac glycosides, steroids, tannins, alkaloids, anthroquinone and terpenoids. *F. proliferatum* SaR-2 and *A. alternata* SaF-2 also exhibited stronger antioxidant activities by FRAP and DPPH method, having the higher levels of phenol and flavonoid than those of plant root. The total amount of phenol and flavonoid quantified were of 21.75, 20.53 gallic acid equivalent per gram and 8.27 and 7.36 μg/mg of quercetin equivalent respectively. These two endophytic fungi (SaR-2 and SaF-2) were found to have comparable scavenging abilities on both FRAP (1682.21 and 1659.05 μmol/mg, respectively) and DPPH-free radicals (90.14% and 83.25%, respectively, at 0.1 mg/mL). This is the first report about isolation of endophytic fungi from *S. miltiorrhiza* Bge.f.alba and their antioxidant activities.

**Conclusions:**

These results indicate that the endophytic fungi associated with *S. miltiorrhiza* Bge.f. alba can be a potential source of novel natural antioxidants.

**Electronic supplementary material:**

The online version of this article (doi:10.1186/s40529-015-0086-6) contains supplementary material, which is available to authorized users.

## Background

*Salvia miltiorrhiza* Bunge is a well-known medicinal plant, and its root, called “dan shen” in Chinese, is a traditional Chinese herbal drug used for the treatment of various kinds of diseases, especially for cardiovascular and cerebrovascular diseases (Zhou et al. [2012]). *S. miltiorrhiza* Bge.f.alba is a white flowered varietas of *S. miltiorrhiza* Bunge and was present only in Shandong province of China. Studies showed that it had special pharmacological effect on thromboangiitis obliterans (Hao et al. [2009]). The main bioactive constituents in root of two kinds of *S. miltiorrhiza* include lipid-soluble diterpenes (dihydrotanshione I, tanshinone I, cryptotanshinone and tanshinone IIA), and water-soluble phenolic compounds (salvianolic, rosmarinic, salvianic acid and protocatechuic) (Chen and Chen [1999]; Liu et al. [2007]). Previous research on phenolic compounds showed that there were important biological activities such as antioxidant and antithrombotic effects (Lam et al. [2007]; Li [1997]; Yan et al. [2006]; Zhou et al. [2005]). Compared with *S. miltiorrhiza* Bunge, *S. miltiorrhiza* Bge.f.alba has higher phenolic acids contents and higher pharmaceutical values with potential application in pharmaceutical industry (Hao et al. [2009]; Hao et al. [2012]). However, the nature source of *S.miltiorrhiza* Bge.f.alba is now in short supply because of the over-collection of the wild plant. Therefore, it is important to find a substitutable approach to produce the active compounds similar with the host plant to meet the medical demand.

Plant endophytic fungi are symbiotic fungi that inhabit the interior of the healthy tissues of the host plants without causing apparent symptoms of disease (Saikkonen et al. [1998]). They have been found in every plant species examined, and it is estimated to be around over one million endophytic fungi colonizing in plants. Endophytic fungi residing within these plants are able to produce bioactive compounds such as paclitaxel, podophyllotoxin and camptothecine (Aly et al. [2010]), which were also produced by their respective host plants. This is advantageous for us to develop an alternative way for efficiently producing these valuable and scarce bioactive constituents. Thus plant endophytic fungi have been considered to be a novel and promising resource of natural bioactive compouds with extensive application in agriculture, industry and medicine field (Schulz et al. [2002]; Strobel et al. [2004]; Verma et al. [2009]). Many valuable bioactive products from endophytes have been recognized as promising sources of antimicrobial, antioxidant, and anticancer substances (Jayanthi et al., [2011]; Lin et al. [2007]; Sadananda et al. [2011]). These bioactive products could be classified as saponins, phenol, flavonoids, cardiac glycosides, steroids, tannins, alkaloids, anthroquinone and terpenoids (Tan and Zou [2001]; Zhang et al. [2006]).

Increasing evidence showed that reactive oxygen species (ROS) could cause oxidative damage of lipids, proteins, DNA and RNA, eventually enhancing the risk for aging, cardiovascular disease, cancer, atherosclerosis, diabetes, Alzheimer’s disease and other diseases (Finkel and Holbrook [2000]; Lachance et al. [2001]). Therefore, antioxidants are believed to be highly effective radical scavengers in the prevention of this ROS mediated diseases. To date, there is little report about isolation and antioxidant activities of endophytic fungi from *S. miltiorrhiza* Bge.f.alba. The present study, therefore, was carried out to better understand the phytochemicals and antioxidant potential of endophytic fungi from *S. miltiorrhiza* Bge.f.alba.

## Methods

### Plant materials

Nine healthy *Salvia miltiorrhiza* Bge. f. alba were randomly collected in July 2010 from *Salvia miltiorrhiza* cultivation zone at three sites in Taishan Medical University, Shandong Province, China. The current-year roots (4–6 cm × 2–3 cm, length × diameter), stems (8–12 cm × 1–2 cm, length × diameter), leaves and flowers were placed in sterile plastic bags and stored at 4°C until isolation procedure was started.

### Isolation of fungal endophytes

Plant tissues were surface-sterilized using the procedure described by Fisher et al. ([1993]) with minor modification**.** The root, stem, leaf and flower segments were cleaned under running tap water. After air-drying, the cleaned stems and roots were cut into small pieces, and then all the tissues were surface sterilized by immersion in 75% ethanol for 1 min, 5% sodium hypochlorite solution for 3 min and then washed three times in sterile distilled water for 1 min each time. The surface-sterilized samples were cut into small pieces using a sterile blade and placed on sterile potato dextrose agar (PDA) at 28°C. The hyphal tip of endophytic fungus growing out from the plant tissue was cut by a sterile pasture pipette and transferred to PDA plate supplemented with ampicillin (150 mg/L) and streptomycin (100 mg/L) to inhibit the bacterial growth until the mycelium or colony appeared surrounding the segments. The efficiency of surface sterilization procedure was checked for each sterilized plant segment following the imprint method. In addition, to check the presence of surface associated fungi, plant samples without surface sterilization were cultured in the same condition as negative controls. Colonization frequency (CF) was calculated as the total number of plant tissue segments colonized by an endophyte, divided by the total number of incubated segments.

Endophytic fungi from the roots, stems, leaves and flowers of *Salvia miltiorrhiza* Bge. f. alba have been numbered and codified as SaR1- SaR7, SaS1, SaL1 and SaF1- SaF5. All the isolated endophytic fungi have been stored on PDA slants at 4°C and kept at College of Life Sciences, Taishan Medical University, Shandong Province, China.

### Identification of fungal endophytes

The identification procedure of endophytic fungi was based on morphology and molecular methods. The morphological characters included culture characteristics and the morphology of conidia. The molecular method was carried out to characterize some non-sporulating group using the ribosomal internal transcribed spacer (ITS) sequence. Fungal genomic DNA was extracted from fresh mycelia using an SDS extraction protocol described by Plaza et al. ([2004]). The primers ITS1 (5’-TCCGTAGGTGAACCTGCGG-3’) and ITS4 (5’-TCCTCCGCTTATTGATATGC-3’) were used to amplify the ITS region**.** The PCR products were subsequently purified and sequenced in two directions on an ABI 3700 automated sequencer. The resulting sequences were subjected to BLAST searches of the NCBI GenBank database to determine the identity of the fungi.

### Fermentation and preparation of crude extracts

Each isolated endophytic fungus strain was inoculated in potato dextrose liquid medium with 150 rpm shaking at 25°C for 5–7 days. Mycelia and broths were separated by filteration through two layers of cheesecloth. Mycelia were thoroughly washed with sterile distilled water, air-dried in an oven at 60°C, and ground into fine powder. The dried mycelia sample (2 g) was extracted with 50 mL of 95% ethanol for three times. Culture filtrates was treated by rotary evaporation under vacuum and then extracted thrice with a threefold of the volume of 95%(v/v) ethanol. The obtained extracts were filtered by Whatman No.1 filter paper and then concentrated under vacuum at 45°C to yield the crude extracts. As a control, 2 g of plant root power was extracted with 50 mL of 95% ethanol and stored at 4°C till further process.

### Phytochemical screening

Preliminary phytochemical analysis of the crude extracts of fungi and plant root was carried out for the presence of the following metabolites such as saponins, phenol, flavonoids, cardiac glycosides, steroids, tannins, alkaloids, anthroquinone and terpenoids according to standard methods (Devi et al. [2012]; Edeoga et al. [2005]; Maobe et al. [2013]).

### Determination of total phenolic content

Total phenolic contents from endophytic fungi and plant root were respectively measured by Folin-Ciocalteu’s colorimetric method (Taga et al. [1984]) with some modification. Briefly, 100 mg of different ethanol extracts was added to 5 mL of 0.3% HCl in methanol/deionised water (60:40, v/v) respectively. The resulting mixture (100 μL) was added to 2 mL of 2% aqueous sodium carbonate. Then the mixture was incubated for 2 min at room temperature. 100 μL of 50% Folin-Ciocalteu’s reagent was added to treated mixture and incubated for 30 min at room temperature, and absorbance was measured at 750 nm with the spectrophotometer against blank. The total phenol content was calculated on the basis of the standard curve of gallic acid. Phenol contents were expressed as mg of gallic acid equivalents (GAEs) per g of extract.

### Determination of total flavonoid content

Total flavonoid content was estimated by a colorimetric method reported by Barros et al. ([2007]). The extract (250 μL) was mixed with distilled water (1.25 mL) and sodium nitrite solution (5%, 75 μL). After 5 min incubation at room temperature, aluminum chloride solution (10%, 150 μL) was added. After 6 min, sodium hydroxide (1 M, 500 μL) and distilled water (275 μL) were added to the mixture. The solution was mixed well and incubated at 25°C for 30 min. Absorbance was measured at 510 nm against blank. The content of flavonoid was calculated on the basis of the standard curve of quercetin and the results were expressed as mg of quercetin equivalents per g of extract.

### Determination of antioxidant activity

Antioxidant activity of examined extracts was measured using ferric ion reducing antioxidant power (FRAP) and 1,1-diphenyl-2-picryl-hydrazyl (DPPH) assay.

### FRAP assay

FRAP reagents was freshly prepared by mixing 25 mL acetate buffer (300 mM, pH 3.6), 2.5 mL 2,4,6-tris (2-pyridyl)-S-triazine (TPTZ) solution (10 mM TPTZ in 40 mM/L HCl) and 2.5 mL FeCl_3_ (20 mM) water solution. Each sample (150 μL) (0.5 mg/mL) dissolved in methanol was added to 4.5 mL of freshly prepared FRAP reagent and stirred. After 5 min, absorbance was measured at 593 nm, using FRAP working solution as blank (Szőllősi and Szőllősi Varga [2002]; Tomic et al. [2009]). A calibration curve of ferrous sulfate (100–1000 μmol/L) was used and results were expressed in μmol Fe^2+^/mg dry weight extract. The relative activity of the samples was compared with the standards ascorbic acid and butylated hydroxytoluene (BHT).

### DPPH radical assay

The free radical scavenging activities of different extracts were carried out by using the 1,1-diphenyl-2-picryl-hydrazyl (DPPH) assay (Braca et al. [2001]). Crude extract (0.1 mL) was mixed with 3 mL of a 0.004% methanol solution of DPPH. The mixture were reacted in the dark condition for 30 min and the absorbance was determined at 517 nm. The percentage inhibition activity was calculated using the following equation:

DPPH scavenging effect (The free radical scavenging ) = [(Abs_control_–Abs_sample_)/Abs_control_] × 100, Where Abs_control_ is the absorbance of the control reaction, Abs_sample_ is the absorbance of the extract/standard.

### Statistical analysis

Analysis of variance was performed by ANOVA. Means between treatment groups were compared for significance by using Duncan new multiple-range test. P values < 0.05 were considered to be significant and P values < 0.01 to be very significant. All experiments were performed in triplicate (n = 3) and results were reported as means ± standard deviation (SD).

## Results

### Isolation and identification of endophytic fungi

A total of 216 tissue segments (including 54 roots, 54 stems, 54 leaves and 54 flowers) collected from 9 individuals of *S. miltiorrhiza* Bge.f.alba at the three sites were processed, and 14 endophytic fungi isolates were recovered. Of these, the majority (n = 7, CF 12.96%) was recorded from roots, followed by flowers (n = 5, 9.26%), stems (n = 1, 1.85%) and leaves (n = 1, 1.85%), respectively (Table 1). 4 isolates mainly belonging to *Alternaria* sp. and Sterile mycelia were isolated from Site 1, 6 strains (*Alternaria* sp., *Fusarium* sp., and Sterile mycelia) were isolated from Site 2 and 4 strains (*Alternaria* sp., and Sterile mycelia) were isolated from Site 3. Among which, *Alternaria* sp. were the dominant species isolated from leaves and flowers, Sterile mycelia occurred in roots and stems, and *Fusarium* sp. were only present in roots. Six isolates of non-sporulating Sterile mycelia were grouped into two morphotypes (morphotypes SaR3, SaR4, SaR5, SaS1, and morphotypes SaR6, SaR7), which were both *Basidiomycota* as indicated by promoting sporulation. Moreover, the selected two non-sporulating fungi (SaR-3, SaR-6), *Alternaria* sp. (SaF-2), and *Fusarium* sp. (SaR-2) were further identified by the internal transcribed spacer (ITS) rRNA gene sequence analysis. The accession numbers were provided by the Genbank. The result of identification showed that two non-sporulating fungi SaR-3 (JQ409156) and SaR-6 (JQ409166) were closely related to *Schizophyllum commune* (EU530002) and *Trametes hirsuta* (EF546240) (Table 2), while SaF-2 (JQ409154) and SaR-2 (JQ409155) were closely related to *Alternaria alternata* (FJ228163) and *Fusarium proliferatum* (EF546240) (Table 2).

**Table 1:** **Genera and number of endophytic fungi recovered from root, stem, leaf and flower of** ***S.miltiorrhiza*** **Bge. f. alba**

Species	Number of isolates and Colonization frequency (%)			
	Sample tissues			
	Root	Stem	Leaf	Flower
	N (%)	N (%)	N (%)	N (%)
*Alternaria* sp.	0 (0)	0 (0)	1 (1.85)	5 (9.26)
*Fusarium* sp.	2 (3.70)	0 (0)	0 (0)	0 (0)
Sterile mycelia	5 (9.26)	1 (1.85)	0 (0)	0 (0)
Total	7 (12.96)	1 (1.85)	1 (1.85)	5 (9.26)

**Table 2:** **Identification of endophytic fungi based on sequence of ITS DNA**

Isolate	Accession number	Size of ITS amplicon (bp)	Closest match in GenBank	Percentage identity
SaF-2	JQ409154	532	*Alternaria alternata*	100
			(FJ228163)	
SaR-2	JQ409155	518	*Fusarium proliferatum*	99
			(EF546240)	
SaR-3	JQ409156	598	*Schizophyllum commune*	99
			(EU530002)	
SaR-6	JQ409166	624	*Trametes hirsuta*	99
			(EF546240)	

### Preliminary phytochemical screening

Preliminary phytochemical screening of fungal ethanolic extracts showed the presence of saponins, flavonoids, cardiac glycosides, terpenoids, steroids, tannins, phenol, anthroquinone and alkaloids. The result of qualitative analysis of the phytochemicals were summarised in Table 3. *A. alternata* SaF-2 and *F. proliferatum* SaR-2 showed presence of all of nine phytochemicals above, while *S. commune* SaR-3, *T. hirsuta* SaR-6 and plant extracts also had all the phytochemical except cardiac glycosides and anthraquinones. Cardiac glycosides was found to be present in the extracts of SaL-1, SaF-1, SaF-2, SaR-1, SaR-2, SaR-5, SaR-6 and SaS-1, while anthroquinone was found to be present in the extracts of SaF-2, SaF-3, SaR-2, SaR-5, SaR-6 and SaR-7. The presence of terpenoids and phenol was observed in all of the endophytes except *Alternaria* sp. SaF-5 and *Schizophyllum* sp. SaS-1, while flavonoids were found to be present in all the extracts except *Alternaria* sp. SaF-1 and *Trametes* sp. SaR-7. The results of phytochemical analysis indicated that the endophytic fungi from *S. miltiorrhiza* Bge.f.alba could produce the same or similar phytochemicals as those originally from the host plant, and new bioactive compounds that were not present in host plant.

**Table 3:** **Phytochemical analysis for the ethanol extracts of different endophytic fungi and plant root**

Genera	Isolates	A	B	C	D	E	F	G	H	I
*Alternaria* sp.	SaL1	-	+	+	+	+	+	+	-	-
	SaF1	+	-	+	+		+	+	-	-
	SaF2	+	+	+	+	+	+	+	+	+
	SaF3	-	+	-	+	+	-	+	+	-
	SaF4	-	+	-	+	-	-	+	-	-
	SaF5	+	+	-	-	-	+	-	-	+
*Fusarium* sp.	SaR1	-	+	+	+	-	+	+	-	-
	SaR2	+	+	+	+	+	+	+	+	+
*Schizophyllum* sp.	SaR3	+	+	-	+	+	+	+	-	+
	SaR4	-	+	-	+	-	+	+	-	-
	SaR5	+	+	+	+		+	+	+	-
	SaS1	-	+	+	-	+	-	-	-	+
*Trametes* sp.	SaR6	+	+	+	+	+	-	+	+	-
	SaR7	-	-	-	+		+	+	+	-
Plant root		+	+	-	+	+	+	+	-	+

+: Presence, −: absence; Sa: *Salvia miltiorrhiza* Bge.f.alba; SaR, SaS, SaL and SaF: isolate assignment code from root, stem, leaf, and flower tissues respectively; A: Saponins, B: Flavonoids, C: Cardiac glycosides, D: Terpenoids, E: Steroids, F: Tannins, G: Phenol, H: Anthroquinone, I: Alkaloids. Data is three replicates of each sample.

### Determination of total phenol content and total flavonoid content

The total phenolic content and total flavonoid content of four endophytic strains (*A. alternata*, *F. proliferatum*, *S. commune* and *T. hirsuta*) and host plant were expressed in terms of mg gallic acid equivalent (GAE)/g of extract and mg quercetin equivalents/g of extract respectively. The higher total phenolic content was found in the extract of *F. proliferatum* SaR-2 (21.75), followed by *A. alternata* SaF-2 extract (20.53) and Plant root extract (19.17) (Table 4). Similarly, the total flavonoid content was found to be higher in *F. proliferatum* SaR-6 (8.27), followed by *A. alternata* SaF-2 (7.36) and plant root (6.98) (Table 4). The more phenol and flavonoid content in the endophytic fungi than host plant may have contributed dramatically to their antioxidant activities.

**Table 4:** **Total antioxidant activity and total phenolic content of ethanol extracts of four endophytic fungi and plant root**

Samples	Phenol (mg/g)	Flavonoid (mg/g)	FRAP (μmol/L)
*Alternaria alternata* SaF-2	20.53 ± 0.08	7.36 ± 0.09	1659.05 ± 0.06
*Fusarium proliferatum* SaR-2	21.75 ± 0.11	8.27 ± 0.12	1682.21 ± 0.05
*Schizophyllum commune* SaR-3	12.96 ± 0.18	5.62 ± 0.15	586.65 ± 0.13
*Trametes hirsuta* SaR-6	11.21 ± 0.25	4.56 ± 0.08	512.25 ± 0.15
Plant root extract	19.17 ± 0.09	6.98 ± 0.13	1347.54 ± 0.11
Ascorbic acid			1655.25 ± 0.07
BHT			66.43 ± 0.16

Data represent means of three replicates.

### Antioxidant capacity analysis

The antioxidant capacity of the ethanol extracts of four selected endophytes and plant extract were assessed for their ability to reduce TPRZ-Fe (III) complex to TPTZ-Fe (II) (Table 4). The reducing ability of the ethanol extracts was in the range of 512.25 to 1682.21 μmol Fe (II)/mg. The FRAP values for the extracts of *F. proliferatum* SaR-2 and *A. alternata* SaF-2 were significantly higher than those of ascorbic acid and BHT, while the FRAP values for the extracts of *S. commune* SaR-3 and *T. hirsuta* SaR-6 were significantly lower than that of ascorbic acid but higher than that of BHT.

**Figure 1:**
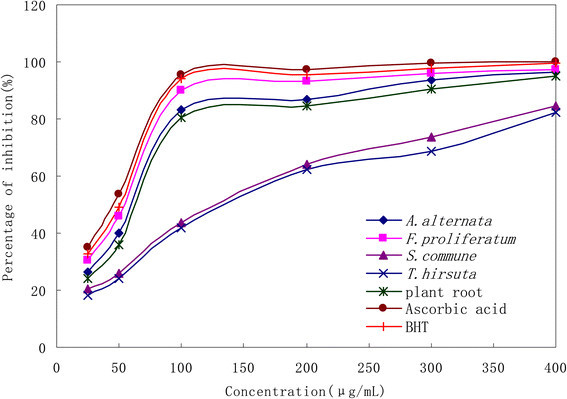
**DPPH radical scavenging activity of the ethanol extracts of four selected endophytic fungi, plant root and the positive controls (ascorbic acid and BHT).** All the experiments were repeated for three times.

The antioxidant activities of the extracts of endophytic fungi and plant root, as compared with the standards ascorbic acid and BHT, were determined by the capability to scavenge DPPH free radicals. Figure 1 showed the dose response curve of DPPH radical scavenging activity of all samples. It was observed that the ethanol extracts of *F. proliferatum* SaR-2 and *A. alternata* SaF-2 had higher radical scavenging activity than that of plant root. At a concentration of 0.1 mg/mL, the scavenging activity of the plant root extract reached 80.23%, while the scavenging activities of *F. proliferatum* SaR-2 and *A. alternata* SaF-2 were 90.14% and 83.25% respectively. Even though the DPPH radical scavenging abilities of the extract of *F. proliferatum* SaR-2 (90.14%) was lower than those of the standards ascorbic acid (95.43%) and BHT (94.14%) at 0.1 mg/mL, it still reached at high concentration. Thus the study showed that *F. proliferatum* SaR-2 could be a promising resource of natural antioxidants.

## Discussion

During this study, an assessment was performed for the diversity of endophytic fungi from *Salvia miltiorrhiza* Bge.f.alba, which are only distributed in Shandong province of China and have been used for the treatment of various cardiovascular diseases (Zhou et al. [2012]). A total of fourteen fungal endophytes were isolated and identified by the morphological and molecular method. Morphological investigations have resulted in the identification of two fungal species: *Alternaria* and *Fusarium* species*.* Six isolates belonging to non-sporulating fungi were identified on the basis of internal transcribed spacer (ITS) rRNA gene sequence analysis, which was consistent with the other reports from different hosts (Liu et al. [2007]). The present study is the first report that several fungal endophytes are associated with *S. miltiorrhiza* Bge.f.alba.

It is interesting to record the predominance of *Alternaria* sp. from this host, as it was also dominant in many other reports from different host plants (Khan et al. [2010]; Kumar et al. [2011]; Liu et al. [2007]). Besides *Alternaria* sp., other genera like *Fusarium* sp. were also reported frequently from a number of other plant species (Liu et al. [2007]; Wang et al. [2008]). Therefore, it appears that *Alternaria* and *Fusarium* species are able to associate endophytically with a wide range of host plants.

Phytochemical analysis was carried out to assess the diversity of chemical compounds produced by the endophytic fungi from *S. miltiorrhiza* Bge.f.alba. The results showed that there were significant amount of secondary metabolites including saponins, flavonoids, terpenoids, steroids, tannins, phenol, and alkaloids in the ethanolic extracts of the endophytic fungi, similar to or with more activity than those in the host plant root extracts. This result was consistent with the earlier reports (Govindappa et al. [2011]; Sadananda et al. [2011]). However, cardiac glycosides and anthraquinones were not present in the plant extracts but in the extracts of *A. alternata* SaF-2, *F. proliferatum* SaR-2, *Schizophyllum sp.* SaR-5 and *T. hirsuta* SaR-6. This result indicated that the fungal endophytes associated with *S. miltiorrhiza* Bge.f.alba had the capability of producing the same or similar phytochemicals as those present in the host plant, and new bioactive compounds that were not present in host plant. It is interesting to find that *Fusarium* sp. and *Alternaria* sp. could produce bioactive compounds originally from their host plants. Similarly, *Fusarium* sp. and *Alternaria* sp. were found to produce active constituents such as paclitaxel, podophyllotoxin, camptothecine, which were also produced by their host plants (Zhao et al. [2011]).

The presence of phytochemicals within fungal endophytes can be promising sources for medicinal or agrochemical use. The phytochemicals including phenols and flavonoids in fungal endophytes may be responsible for the antioxidant property (Hamilton et al. [2012]). According to recent study, a highly positive correlation between total phenol content, total flavonoid content and antioxidant activity seems to be the trend in many endophytic fungi. In our study, strong antioxidant activities were present in the ethanol extracts of *F. proliferatum* SaR-2 and *A. alternata* SaF-2, comparable with the standards ascorbic acid and BHT. High phenolic and flavonoid content found in the ethanol extracts of *Fusarium* sp. and *Alternaria* sp. imply the contribution of these compounds to antioxidant activities, which was consistent with early study (Govindappa et al. [2011]; Murthy et al. [2011]; Sadananda et al. [2011]). These findings indicate that endophytic fungi from *S. miltiorrhiza* Bge.f.alba may be effective as a promising potential for the development of novel antioxidant drugs. However, further studies are recommended to purify and characterize the structure of the biologically active constituents.

## Conclusions

The present study demonstrated that fungal endophytes, *Alternaria alternata* SaF-2 and *Fusarium proliferatum* SaR-2 from *Salvia miltiorrhiza* Bge.f.alba yielded medically important phytochemical compounds. The antioxidant potential may be directly linked to the phenolic and flavonoid compounds present in the ethanol extracts of *A. alternata* SaF-2 and *F. proliferatum* SaR-2. To our knowledge, this is the first report that fungal endophytes associated with *S. miltiorrhiza* Bge.f.alba have been found to possess antioxidant potential.

## Authors’ original submitted files for images

Below are the links to the authors’ original submitted files for images. Authors’ original file for figure 1

## Abbreviations

BHT: Butylated hydroxytoluene
CF: Colonization frequency
DPPH: 1,1-diphenyl-2-picryl-hydrazyl
FRAP: Ferric ion reducing antioxidant power
GAEs: Gallic acid equivalents
ITS: Internal transcribed spacer
mg/g: Milligram/gram
PDA: Potato dextrose agar
ROS: Reactive oxygen species
Sa: *Salvia miltiorrhiza* Bge.f.alba
SaF: SaL, SaR and SaS, Flower, Leaf, Root and Stem tissues of *Salvia miltiorrhiza* Bge.f.alba
TPC: Total Phenolic Content
TPTZ: 2,4,6-tris (2-pyridyl)-S-triazine
