# Supplementary figures and images for: The endophytic fungi of Salvia miltiorrhiza Bge.f. alba are a potential source of natural antioxidants
Source: Bot Stud. 2015 Apr 1;56:5. doi: 10.1186/s40529-015-0086-6 (PMC5430307; doi:10.1186/s40529-015-0086-6)

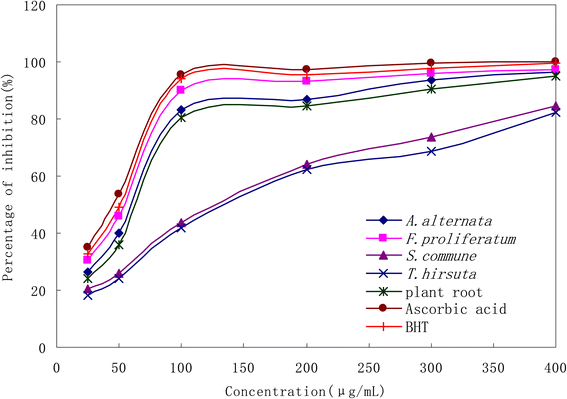

Supplement: Supplementary file 1 — Authors’ original file for figure 1 [file 40529_2015_86_MOESM1_ESM.gif]
